# Subjective Well-Being among Primary Health Care Patients

**DOI:** 10.1371/journal.pone.0114496

**Published:** 2014-12-08

**Authors:** Alis Ozcakir, Fatma Oflu Dogan, Yakup Tolga Cakir, Nuran Bayram, Nazan Bilgel

**Affiliations:** 1 Uludag University Faculty of Medicine, Department of Family Medicine, Bursa, Turkey; 2 Uludag University Faculty of Economics and Administrative Sciences, Department of Econometrics, Bursa, Turkey; University of New South Wales, Australia

## Abstract

**Background:**

The psychological importance of subjective well-being for a healthy life has been well recognized. It is also well known that depressive and anxiety disorders have a negative effect on subjective well-being. The aim of this cross-sectional, descriptive study was to assess the subjective well-being status of a group of primary healthcare patients in relation to socio-demographic characteristics, personal health and mood-status.

**Methods:**

A total of 284 patients participated in the study. The Oxford Happiness Scale, Life Satisfaction Scale, DASS-42 (Depression, Anxiety and Stress Scales-42) and a questionnaire about socio-demographic characteristics were completed by the participants.

**Results:**

In general, the participants were found to be moderately happy and satisfied with their lives. They had mild levels of depression, anxiety and stress. In terms of happiness, an older age (≥40 years), educated to secondary level or higher and not having depression or anxiety were found to be factors increasing happiness. In terms of life satisfaction, female gender, an older age (≥40 years), educated to secondary level or higher, being single and not having depression were found to increase life satisfaction.

**Conclusion:**

Primary healthcare providers should give more importance to the mood status of their patients. Screening for depression and anxiety should be applied at the primary healthcare level because negative mood status is more important than some socio-demographic characteristics in respect of unhappiness and dissatisfaction.

## Introduction

Over the past 40 years, researchers have tried to define subjective well-being and explain its correlations and consequences. Subjective well-being, which is synonymous with happiness, psychological well-being or mental well-being is attracting increasing attention in the field of positive psychology [1], [2], [3], [4]. Much of what we know about subjective well-being is based on the findings of a great number of representative surveys that have asked participants to report how happy and satisfied they are with their life as a whole and with various life domains. Happiness can be described as often being in a state of joy, or as a state of satisfaction. Being in a state of joy is an emotion and being satisfied is cognition. Measures of subjective well-being emphasize both emotions and cognitions.

In previous studies, many variables have been shown to be related to subjective well-being. In respect of socio-demographic characteristics for example, education, wealth and being married have been determined to be positively related to happiness whereas age has been found to be related to satisfaction but not to happiness because older people experience emotions less intensely than younger people [3], [5]. Among the many factors which affect subjective well-being and happiness, being healthy is also important. On the other hand, many studies have shown that subjective well-being protects individuals from both physical and psychological disorders. Studies have shown that happiness appears to foster physical health and high optimism prevents cardio-vascular diseases and death [6], [7], [8], [9]. Optimism and positive emotions have also been linked to faster recovery rates and to a greater adherence to the medical regimen [10], [11]. Thus subjective well-being may act as a preventive factor [12], [13], [14], [15]. Happiness and life satisfaction predicted lower risk of all-cause mortality in healthy populations [16]. Furthermore life satisfaction, absence of negative emotions, optimism, and positive emotions have been reported to result in better health and longevity [17], [18], [19].

It is also well known that depressive and anxiety disorders have a negative effect on subjective well-being. Researchers have reported that the severity of anxiety is associated with significant impairments in psychological well-being and the presence of a depressive disorder comorbid with an anxiety disorder had a negative impact on quality of life and life satisfaction [20], [21], [22]. Positive psychological interventions have been seen to decrease depression and pain among primary health care patients [23]. Mood disorder and impaired emotional and social role functioning have been found to be associated with unhappiness [24].

In Turkey, the subjective well-being of the general population and of patients is a neglected issue and studies on this subject are rare. The studies available on life satisfaction and happiness for the general population in Turkey used the data sets of the World Values Survey and World Database of Happiness [25], [26]. In these studies which were conducted on the general population, the areas of happiness and life satisfaction were assessed with only one question of: “Are you happy” or “Are you satisfied with your life?” The results of these studies have shown discrepancies. One study found a significant negative effect of age on happiness and life satisfaction whereas the other study determined a positive significant effect [27], [28]. One study showed that being male has a significantly negative direct effect on happiness whereas the other study found that gender had no effect [27], [28]. Furthermore, one study found no significant effect of education whereas the other study revealed a significant positive relationship between education and happiness [27], [28]. Both of these studies showed a significant positive relationship between happiness, life satisfaction and higher levels of income. Compared to previous studies in Turkey, the current study is of importance because the data were collected directly from the participants. No previously collected data were used and the assessment of subjective well-being was made using validated scales.

The purpose of this study was to assess the subjective well-being status of a group of patients who attended a primary healthcare unit in Turkey. Of the many factors which have been found to be related to subjective well-being evaluations were made to assess relationships between:

1. Some socio-demographic characteristics (age, sex, education, marital status and income) and subjective well-being;
2. Having a chronic disease and subjective well-being;
3. Experiencing any kind of loss (family member, money or job) and subjective well-being;
4. Mood status (depression, anxiety, stress) and subjective well-being.

## Materials and Methods

### Study design

This was a cross-sectional, descriptive study which depended on self-reporting.

### Ethical Issues

Approval for the study was granted by Uludag University Faculty of Medicine Ethics Committee (Date of approval: 31 July 2012; number: 2012-17/2). The study was conducted in accordance with the Declaration of Helsinki. Written informed consent forms were seen and approved by the Uludag University Faculty of Medicine Ethics Committee during the approval process of the study. All participants gave written informed consent before taking part and the informed consent forms were collected in a separate file.

### Place of the study

This study was performed in a primary healthcare unit in Bursa, Turkey. This primary healthcare unit is affiliated to the medical faculty and serves as a training center for medical students and research assistants of the Family Medicine Department.

### Study participants

During a period of two months, 378 adult patients (aged 18 years and over) attended this unit. All were asked to participate in the study after the neccessary information about the study was given. Written informed consent was obtained from 284 patients and 94 patients did not want to participate. The response rate to the study was 75.1%.

### Study materials

All of the study materials were printed materials, which were distributed to the patients who then answered the questions anonymously. The printed materials used were as follows:

1. A questionnaire about the socio-demographic characteristics of the participants such as, sex, age, marital status, educational attainment and income. Two further questions were asked on this questionnaire: “Have you been diagnosed or treated for clinical depression during the last year?” “Have you experienced any kind of loss (family member, money or job) during the last year?
2. The Turkish version of the Oxford Happiness Questionnaire (OHQ): The original scale was developed by Hills and Argyle, adapted for Turkey by Seker and Gencdogan and the psychometric analyses of the Turkish version were made by Dogan and Sapmaz [29], [30], [31]. The Turkish form of OHQ has a one-factor structure. The Cronbach alpha internal consistency coefficient of OHQ in Turkish was 0.91, the reliability coefficient obtained with test half-life method was 0.86 and the composite reliability of the scale was 0.91 [31]. The Turkish version of the OHQ is similar to the original and has 29 single items that respondents may answer on a uniform six-point Likert scale. The sum of the item scores is an overall measure of happiness, with high scores indicating greater happiness. The range of the total item scores is 29-174 points.
3. The Turkish version of the Satisfaction with Life Scale (SWL): The original scale was developed by Diener, Emmons, Larsen and Griffin, adapted for Turkish by Koker and psychometric analyses were made by Yetim [32], [33], [34]. The Cronbach alpha internal consistency coefficient of SWL in Turkish was found to be 0.76 and test re-test reliability was 0.85 [34]. The Turkish version of the SWL scale is similar to the original and has 5 single items that respondents answer on a uniform seven-point Likert scale. The sum of the item scores is an overall measure of life satisfaction, with high scores indicating greater life satisfaction. The range of the total item scores is 5-35 points.
4. Depression, Anxiety and Stress Scales-42 (DASS-42). The original scale was developed by Lovibond and Lovibond [35]. The DASS-42 is a 42-item instrument measuring current (within the past week) symptoms of depression, anxiety, and stress. Each of the three scales consists of 14 items answered using a 0-3 scale, where 0 = did not apply to me at all, and 3 = applied to me very much or most of the time (range of possible scores for each scale is 0–42). Scores considered in the normal range are 0–9 for depression, 0–7 for anxiety, and 0–14 for stress. Scores above these ranges indicate the degree of the problem from mild to extreme. Psychometric analyses of the Turkish version of DASS-42 were performed by Bilgel and Bayram [36]. The Cronbach alpha internal consistency coefficients of DASS-42 in Turkish were found to be 0.92, 0.86, and 0.88 for depression, anxiety, and stress, respectively. Construct validity measured by item-scale correlations ranged from 0.48 to 0.70 for depression, from 0.33 to 0.59 for anxiety, and from 0.43 to 0.70 for stress. DASS-42 in Turkish showed a good convergent validity and a three factor structure like the original scale.

### Study statistics

The IBM SPSS Statistics 22 program was used for descriptive statistics, chi square analyses, t-tests, ANOVA, correlation and multiple regression analyses.

## Results

The Cronbach alpha internal consistency coefficients of the scales for the study group were as follows: OHQ  = 0.92; SWL = 0.88; DASS-42- Depression = 0.95, Anxiety = 0.92 and Stress = 0.93.

The study group consisted of 284 participants; 51.1% were female and 65.8% were aged 39 years and below. The socio-demographic characteristics of the study group are shown in Table 1.

**Table 1:** Socio-demographic characteristics of the study group.

	Female N (%)*	Male N (%)*	Total N (%)**	χ^2^	p
AGE					
≤39	96 (51.3)	91 (48.7)	187 (65.8)	0.017	0.896
≥40	49 (50.5)	48 (49.5)	97 (34.2)		
MARITAL STATUS					
Single	56 (51.4)	53 (48.6)	109 (38.4)		
Married	81(51.6)	76 (48.4)	157 (55.3)	0.337	0.845
Other	8 (44.4)	10 (55.6)	18 (6.3)		
EDUCATION					
Primary	40 (61.5)	25 (38.5)	65 (22.9)		
Secondary	30 (38.5)	48 (61.5)	78 (27.5)	8.067	0.018
Higher	75 (53.2)	66 (46.8)	141 (49.6)		
INCOME (monthly, US$)					
≤328	38 (58.5)	27 (41.5)	65 (22.9)		
329–657	57 (50.0)	57 (50.0)	114 (40.1)		
658–985	22 (51.2)	21 (48.8)	43 (15.1)	3.196	0.525
986–1314	14 (51.9)	13 (48.1)	27 (9.5)		
≥1315	14 (40.0)	21 (60.0)	35 (12.3)		

* % within rows ** % within columns.

Except for educational level, no statistically significant differences were found between male and female participants in terms of socio-demographic characteristics. Descriptive statistics for total happiness, depression, anxiety, stress and life satisfaction scores are shown in Table 2.

**Table 2:** Mean and median values for OHQ, SWL and DASS-42.

	Mean	Median	Standard Deviation	Standard Error
Oxford Happiness Scale	116.04	119.00	22.49	1.33
Satisfaction with Life Scale	22.06	23.00	7.85	0.46
DASS-42 Depression	8.89	6.00	8.79	0.52
DASS-42 Anxiety	9.16	8.00	7.98	0.47
DASS-42 Stress	13.65	13.00	9.15	0.54

The study group was moderately happy and moderately satisfied with their lives. However, although mean depression and stress scores were within the normal range (0–9 for depression and 0–14 for stress) they were very close to the threshold values. Furthermore, it can be said that this study group was anxious with a mean anxiety score of 9.16 which is higher than the normal range (0–7).

Of the total participants, 46 (16.2%) reported that they had been diagnosed with clinical depression and received treatment during the last year. Furthermore, 73 (25.7%) participants reported that they had experienced the loss of a family member, money or job during the last year. Some participants had a diagnosis of a chronic disease, such as diabetes, cardio-vascular diseases, hypertension, neurological disorders or osteo-arthritis. The results of the comparison of the happiness, life satisfaction, depression and anxiety scores of the participants who had reported the above-mentioned states compared to those who had not are shown in Table 3.

**Table 3:** The subjective well-being and depression anxiety stress status of participants who were in special circumstances during the last year compared to those who were not.

Special circumstance at last year	OHQ Score	SWL Score	Depression	Anxiety	Stress
	Mean±SE	Mean±SE	Mean±SE	Mean±SE	Mean±SE
Depression					
Yes	98.95±4.00	18.86±1.27	17.04±1.40	16.91±1.35	21.56±1.29
No	119.34±1.29	22.68±0.48	7.31±0.50	7.66±0.44	12.12±0.54
t-test	p<0.001	p<0.001	p<0.001	p<0.001	p<0.01
Loss					
Yes	111.77±3.12	19.16±0.89	10.76±1.16	11.23±1.08	15.34±1.28
No	117.52±1.42	23.06±0.53	8.24±0.57	8.45±0.51	13.07±0.57
t-test	N.S.	p<0.001	N.S.	p<0.05	N.S.
Chronic disease					
Yes	110.49±3.15	20.73±0.92	10.51±1.19	10.35±1.01	15.74±1.14
No	118.14±1.37	22.57±0.54	8.28±0.56	8.71±0.53	12.86±0.61
t-test	N.S.	N.S.	N.S.	N.S.	p<0.05

Participants who were diagnosed with clinical depression in the last year had significantly lower happiness and life satisfaction scores than those without such a diagnosis. They also had significantly higher depression, anxiety and stress scores. Participants who reported any kind of loss had significantly lower life satisfaction scores, whereas happiness, depression, anxiety and stress scores did not differ from the participants without any loss. Participants with chronic disease were found to be more stressed than those without any chronic disease but no significant differences were found in terms of happiness, life satisfaction, depression and anxiety scores.

The mean OHQ, SWL scores according to the socio-demographic characteristics and mood status of the participants are shown in Table 4.

**Table 4:** Mean OHQ, SWL scores according to socio-demographic characteristics and mood status.

		OHQ	SWL
SOCIO-DEMOGRAPHICS		Mean ± SE	Mean ± SE
GENDER	Male	117.67±1.99	21.69±.64
	Female	114.47±1.78	22.41±.67
Test Statistics & p value	t-test	1.200; p = 0.23	0.768; p = 0.44
AGE	≤39	114.19±1.73	21.39±.58
	≥40	119.60±1.96	23.35±.77
Test Statistics & p value	t-test	1.93; p = 0.05	2.00; 0.05
MARITAL STATUS	Single	118.97±1.95	23.49±.68
	Married	116.04±1.69	21.92±.63
	Other	98.33±8.45	14.61±1.85
Test Statistics & p value	ANOVA	6.77; p = 0.001	10.63; p = 0.001
EDUCATION	Primary	101.07±3.32	17.83±1.19
	Secondary	118.61±2.17	22.89±.85
	Higher	121.52±1.58	23.55±.54
Test Statistics & p value	ANOVA	21.89; p = 0.001	13.53; p = 0.001
INCOME (Monthly US$)	≤328	109.83±3.58	21.57±1.04
	329–657	112.46±1.78	20.32±.74
	658–985	124.93±2.42	24.91±1.18
	986–1314	119.52±4.40	24.22±1.51
	≥1315	125.63±3.40	23.51±.89
Test Statistics & p value	ANOVA	5.75; p = 0.001	3.85; p = 0.005
MOOD STATUS			
Depression	None	126.06±1.27	25.06±0.49
	Mild	113.79±2.35	20.95±1.26
	Moderate	102.24±2.32	18.15±0.89
	Severe	95.50±4.28	15.36±2.06
	Extremely severe	69.07±6.42	8.93±0.98
Test Statistics & p value	ANOVA	52.22; p = 0.001	32.21; p = 0.001
Anxiety	None	125.31±1.55	24.86±0.57
	Mild	120.31±2.37	23.75±1.16
	Moderate	107.35±2.58	19.31±0.88
	Severe	108.21±3.48	19.43±1.59
	Extremely severe	90.63±5.49	14.45±1.54
Test Statistics & p value	ANOVA	24.52; p = 0.001	17.42; p = 0.001
Stress	None	125.07±1.37	24.79±0.51
	Mild	112.29±2.46	20.56±1.16
	Moderate	103.30±2.86	18.85±1.06
	Severe	93.71±5.52	15.85±1.71
	Extremely severe	90.71±11.76	11.00±1.94
Test Statistics & p value	ANOVA	26.10; p = 0.001	18.61; p = 0.001

Strong significant and positive correlations were determined between happiness and life satisfaction. Correlations between happiness and depression, anxiety and stress were strong, significant and negative. Similar correlations were found for life satisfaction. Furthermore, strong and positive correlations were found between depression, anxiety and stress.

Multiple regression analyses were applied to assess the impact of socio-demographic variables, factors like having a chronic disease and experiencing any kind of loss and depression, anxiety and stress on happiness and life satisfaction (Table 5 and Table 6).

**Table 5:** The effects of socio-demographic variables, and mood status on happiness.

HAPPINESS							
MODEL A				MODEL B			
VARIABLES	Beta	t	p	VARIABLES	Beta	t	p
Gender	0.022	0.412	N.S.	Gender	−0.041	−1.023	N.S.
Age	0.291	3.982	<0.001	Age	0.201	3.718	<0.001
Education	0.336	5.160	<0.001	Education	0.254	5.265	<0.001
Marital status	−0.170	−2.259	<0.05	Marital status	−0.111	−2.009	N.S.
Income	0.174	2.959	<0.01	Income	0.075	1.688	N.S.
				Loss	−0.048	−1.175	N.S.
				Chronic disease	−0.026	−0.619	N.S.
				Depression	−0.682	−8.626	<0.001
				Anxiety	0.192	2.433	<0.05
				Stress	−0.128	−1.704	N.S.
F	13.33			F	36.42		
p	<0.001			p	<0.001		
Adjusted R^2^	0.18			Adjusted R^2^	0.56		

**Table 6:** The effects of socio-demographic variables, and mood status on life satisfaction

LIFE SATISFACTION							
MODEL A				MODEL B			
VARIABLES	Beta	t	p	VARIABLES	Beta	t	p
Gender	−0.081	−1.434	N.S.	Gender	−0.136	−2.870	<0.01
Age	0.268	3.529	<0.001	Age	0.189	2.962	<0.01
Education	0.257	3.797	<0.001	Education	0.187	3.290	<0.01
Marital status	−0.222	−2.841	<0.01	Marital status	−0.174	−2.669	<0.01
Income	0.108	1.772	N.S.	Income	0.018	0.349	N.S.
				Loss	−0.164	−3.507	<0.001
				Chronic disease	0.001	0.024	N.S.
				Depression	−0.527	−5.662	<0.001
				Anxiety	0.116	1.250	N.S.
				Stress	−0.135	−1.523	N.S.
F	8.06			F	20.45		
p	<0.001			p	<0.001		
Adjusted R^2^	0.11			Adjusted R^2^	0.40		

In Model A, only the socio-demographic variables were included in the analyses both for happiness and life satisfaction and these variables explained 18% and 11% of the variance in happiness and life satisfaction respectively. In Model B, factors like having a chronic disease and experiencing any kind of loss and depression, anxiety and stress scores were added to the model. Socio-demographic variables and the newly added variables together explained 56% of happiness and 40% of life satisfaction. When the results of Model B were taken into account, happiness was found to be related to age (participants ≥40 years of age were happier than those ≤39 years of age), educational level (participants with secondary and higher education were happier than those with only primary education), depression and anxiety levels (participants without depression and anxiety were happier than participants with different levels of depression and anxiety). Gender, marital status, income, having a chronic disease, experiencimg any kind of loss and stress levels were not found to be related to happiness.

According to the results of Model B, life satisfaction was found to be related to gender (female participants were more satisfied than males), age (participants ≥40 years of age were more satisfied than those ≤39 years of age), educational level (participants with secondary and higher education were more satisfied than those with only primary education), marital status (single participants were more satisfied), and depression levels (participants without depression were more satisfied than participants with different levels of depression). Furthermore participants who did not experience any kind of loss were more satisfied with their lives than those with such an experience. Income, anxiety stress and having a chronic disease were not found to be related to life satisfaction.

## Discussion

In this study, the subjective well-being of a group of primary care patients was assessed in terms of life satisfaction and happiness. As mentioned in the Introduction, subjective well-being is an important component of being healthy and also has a strong effect on healing, therefore this issue should not be neglected in primary healthcare service. There have been several previous studies in Turkey concerning life satisfaction and happiness, most of which have been conducted on students, adolescents and the elderly [37], [38]. Studies which are representative of the Turkish general population have mostly dealt with economic issues and subjective well- being [27], [28]. In a study by Selim, the effect of age on happiness and life satisfaction was found to be negative and it was concluded that individuals in older age-groups are less happy and less satisfied than individuals in the youngest age-group (15–24 years) [27]. A similar result was reported by Haller and Hadler [39]. Another study found a positive relationship between satisfaction, happiness and age which is in line with the findings of the current study [28]. Why older people are happier and more satisfied needs to be further studied but could be explained by the experience and wisdom gained throughout life and by diminished expectations. In this study, gender was not found to be related to happiness but to life satisfaction. Female participants were significantly more satisfied than male participants. Women in Turkey have less freedom to make decisions about their way of life compared to many other countries. They are also less involved in the areas of work and public life, and have less power of decision in work organizations and politics. However, all these discriminations do not seem to reduce their life satisfaction. Some studies in the literature also found that being male had a significantly negative direct effect on happiness whereas some found no effect [40], [41], [42], [43]. A previous Turkish study found that females were happier and more satisfied than males and that result is in line with this current study [27]. According to the results of the current study, being married has no significant effect on happiness but there is an effect on life satisfaction, whereas some other studies have found that married people are both happier and more satisfied than single, divorced or widowed individuals [27], [39], [43]. A previous study from Turkey found a positive relationship between happiness and educational level which is consistent with the current study results [28]. Therefore, improving the educational level of the population should be considered a government priority with wider public health implications. No significant relationship was found between income and happiness or life satisfaction, whereas some previous studies have found a positive correlation between economic well-being and happiness and life satisfaction [27], [28], [44]. Some studies in the literature showed the presence of a chronic disease is associated with subjective well-being [45] whereas in this study we did not find such an association. This may be due to several reasons. In our study group only 27.5% of participants reported the presence of a chronic disease and this amount may be not enough for revealing such an association. Furthermore we did not assess the severity of their chronic diseases or perceptions and coping mechanisms against these diseases which may influence subjective well-being. Therefore we think that life satisfaction and happiness among primary health care patients with chronic diseases should be a concern of another study.

The current study showed that mood status has a greater impact on happiness and life satisfaction than socio-demographic characteristics like as gender, age, marital status and income. The relationship between mood status and health outcomes illustrates the need to detect mood disturbance and psychological distress in patients seen in primary health care settings. Screening is important to determine whether patients would be served by an intervention designed to improve mental and social functioning and reduce negative thoughts and mood. Several self-reported screening tools for mood status are available in Turkish, of which the most used are the Beck Depression Inventory II and the Beck Anxiety Inventory. However, the DASS-42 can be considered to be more user-friendly and practical than the other scales. Screening of mood status will enable earlier diagnosis of depression and anxiety by referring those with scores above threshold values to a psychiatrist. Just as in other diseases, primary healthcare providers can also act as gate-keepers for mood disorders. If the burden of depression is considered worldwide (depression is the 4^th^ leading cause of disability worldwide, and is a major contributor to the global burden of diseases. Mental disorders accounted for 25.3% and 33.5% of all years lived with a disability in low- and middle-income countries, respectively) and in Turkey (major depressive and anxiety disorders are among the top five leading causes of years lived with disability), the importance of screening for mood disorders at the primary care level can be more clearly understood [46], [47]. Furthermore, patients with chronic diseases and patients who have recently experienced any kind of loss (family member, money or job) should be monitored by primary healthcare physicians carefully as they are at risk of being unhappy and dissatisfied with their lives.

At the primary healthcare level some other attempts could be made to improve happiness and life satisfaction. There are several examples of such kinds of interventions in the literature, such as the Positive Psychology Interventions (PPI), promoting resilience, optimism, or gratitude, which could be applied at the primary health care level. Skills that can improve individual resilience such as challenging beliefs, avoiding thinking traps, calming and focusing and putting things in perspective could be taught by PPIs [48], [49], [50].The Values in Action Inventory of Strengths (VIA-IS) could also be applied at the primary healthcare level to measure the character strengths of human goodness and excellence. These strengths are known as “signature strengths”, are not bound to culture, but are perceived as natural and desirable with an energizing rather than exhausting effect on individuals [51].

Positive psychology and related interventions are relatively new concepts and unfortunately not well known in Turkey. On the other hand, studies mostly from developed Western countries have shown that these interventions have an enormous positive impact on mental well-being, happiness and life satisfaction. Primary healthcare providers have the opportunity to offer and evaluate positive psychology practices across diverse socio-demographic subgroups that currenly receive little attention. It is hoped that more studies related to life satisfaction and happiness will increase the implementation of PPIs and that through these interventions the subjective well-being of the community will be enhanced.

## Conclusions

The relationship between depression and subjective well-being illustrates the need to detect mood disturbance and psychological distress in patients seen in primary health care settings. Numerous psychometric tools can be used as screening tools and general practitioners can screen to identify individuals likely to have depression. However screening cannot be used independently for the diagnosis of depression and should be followed by clinical diagnostic interview. If resources are available and procedures are in place for accurately diagnosing, treating, referring or following up patients likely to have depression screening may improve subjective well-being.

## Limitations

The limitations of this study include restricted generalization as the study was performed in a single primary healthcare unit.There may also have been recall-bias or incorrect answers as the study depended on self reporting.

## Supporting Information

Data S1 **Data S1 in Excel format.**
(XLS)
